# Changes in Plasma Glycine Concentration in General Anesthesia With Remifentanil: A Prospective Observational Pilot Study

**DOI:** 10.7759/cureus.96727

**Published:** 2025-11-12

**Authors:** Takao Kato, Kikumi Matsuoka, Asako Nagane, Yuki Kurokawa, Yui Yoshida, Kaoru Koyama

**Affiliations:** 1 Department of Anesthesiology, Saitama Medical Center, Saitama Medical University, Kawagoe, JPN; 2 Department of Biomedical Sciences, Saitama Medical Center, Saitama Medical University, Kawagoe, JPN; 3 Department of Anesthesiology, Saitama Sekishinkai Hospital, Sayama, JPN

**Keywords:** general anesthesia, glycine, opioid-induced hyperalgesia, postanesthetic shivering, remifentanil

## Abstract

Background: Remifentanil-based anesthesia has been associated with postanesthetic shivering (PAS) and postoperative pain consistent with opioid-induced hyperalgesia (OIH). PAS and OIH have been suggested to involve N-methyl-D-aspartate (NMDA) receptor signaling. Glycine participating in the NMDA receptor functions as a co-agonist. Commercial remifentanil formulations contain glycine as an excipient, raising the question of whether perioperative exposure to this medication increases circulating glycine levels, potentially including those in the central nervous system.

Objective: The objective of this study is to confirm the elevation of plasma glycine and its perioperative course during general anesthesia with glycine-containing remifentanil.

Methods: An open-label, single-center, preliminary prospective observational study was performed in adults undergoing elective pancreaticoduodenectomy. Patients with renal dysfunction or liver dysfunction were excluded. Balanced anesthesia included desflurane, propofol, remifentanil (Ultiva; 7.5 mg glycine per 1 mg remifentanil) infused at 0-0.2 μg/kg/min, fentanyl, and rocuronium. No glycine-containing IV fluids were administered. Plasma glycine was quantified by enzyme-linked immunosorbent assay at six time points: pre-induction (T0), 120 min after induction (T1), end of remifentanil (T2), and one hour (T3), two hours (T4), and three hours (T5) after completion. PAS, Face Pain Scale-revised (FPS-R), ICU Confusion Assessment Method (CAM-ICU), and ionized magnesium on ICU admission were recorded exploratorily. Changes over time were assessed using a repeated-measures ANOVA with the Greenhouse-Geisser correction and Dunnett comparisons versus T0 (α = 0.05).

Results: Twelve patients were enrolled, and 10 participants completed the protocol. Plasma glycine ranged from 200 to 500 μmol/L. There was no significant increase at any remifentanil infusion rate. Compared with pre-induction, plasma glycine levels were significantly lower at two hours (T4) and three hours (T5) post-remifentanil (p < 0.05). One patient exceeded the reference range for glycine despite preservation of hepatic and renal function. The study was not powered to test associations with PAS or OIH.

Conclusions: In surgical patients with preserved organ function, anesthesia with glycine-containing remifentanil did not elevate plasma glycine levels in 9 of 10 cases, limiting evaluation of an NMDA receptor-mediated mechanism. Further investigation should consider evaluating plasma concentrations when high-concentration remifentanil or glycine-containing formulations are used concomitantly.

## Introduction

Remifentanil-based anesthesia has been associated with higher rates of postanesthetic shivering (PAS) and postoperative pain consistent with opioid-induced hyperalgesia (OIH), compared with alternative opioid regimens [1-6]. Although the underlying mechanisms remain incompletely defined, clinical mitigation with small doses of ketamine and magnesium sulfate plays a role in glutamatergic/N-methyl-D-aspartate (NMDA) receptor pathways in these phenomena [7-9].

Commercial remifentanil contains glycine as an excipient, leading to concomitant systemic glycine exposure during anesthesia [10]. Glycine is synthesized from serine and is cleared primarily via the glycine cleavage system, while also participating in hepatic glycine-conjugation pathways; at NMDA receptors, it functions as an obligatory co-agonist that facilitates receptor activation when extracellular levels rise [11-13]. Consistent with this pharmacology, formulation-derived glycine and remifentanil itself have been linked to NMDA-dependent processes in preclinical studies [14-16]. While glycine crosses the blood-brain and blood-cerebrospinal fluid (CSF) barriers primarily by passive diffusion, its brain uptake is extremely low; active brain-to-blood efflux systems help maintain a low CSF glycine level at baseline [17]. However, marked elevation of plasma glycine (e.g., after intravenous administration) can lead to an increased CSF glycine level in humans [18]. In non-ketotic hyperglycinemia, sustained systemic elevation is accompanied by a high CSF glycine level and neurological toxicity [19]. During prolonged intensive care unit (ICU) sedation with remifentanil, plasma glycine may rise in a dose-dependent manner, particularly in patients with organ dysfunction [20,21].

These observations led us to hypothesize that, even in otherwise healthy surgical patients, a substantial perioperative increase in circulating glycine could elevate central glycine and facilitate NMDA-mediated adverse effects, potentially contributing to PAS/OIH. However, perioperative plasma glycine dynamics during remifentanil-based general anesthesia have not been well characterized. Therefore, the aim of this study was to measure the time course of plasma glycine during remifentanil anesthesia in patients with preserved hepatic and renal function.

## Materials and methods

An open-label, single-center, preliminary prospective observational study was conducted in accordance with the principles in the Declaration of Helsinki and Good Clinical Practice guidelines. With approval of the local ethics committee (authorization number: 1545), written informed consent was obtained from all patients. The study was prepared in accordance with the Strengthening the Reporting of Observational Studies in Epidemiology (STROBE) statement.

The subjects were 12 patients who underwent elective pancreaticoduodenectomy without revascularization at Saitama Medical Center, Saitama Medical University, between January and August 2017. Patients were excluded if they had one of the following conditions: preoperative use of glycine-containing medicine, preexisting renal dysfunction (estimated glomerular filtration rate <60 ml/min), or liver dysfunction (serum total bilirubin >2.0 mg/dl).

All patients underwent balanced anesthesia with desflurane, propofol, remifentanil, fentanyl, and rocuronium. Each 1 mg of remifentanil (Ultiva®) used in the study contains 7.5 mg of glycine. The remifentanil infusion rate was determined at the discretion of the attending anesthesiologist and administered at fixed rates during surgery: not used, 0.05, 0.1, 0.15, or 0.2 μg/kg/min. The remifentanil was discontinued immediately upon completion of surgery. An invasive arterial pressure line (FloTrac Sensor®, Edwards Lifesciences Corp., Irvine, CA) was inserted into the radial artery. Arterial pressure cardiac index (APCI) and stroke volume variation (SVV) were monitored as hemodynamic parameters. If necessary, a central venous catheter was inserted in the internal jugular vein, and epidural anesthesia was performed. Hemodynamics were managed to maintain mean arterial pressure (MAP) ≥65 mmHg, using APCI, SVV, and central venous pressure (CVP, when present) as adjuncts to guide therapy.

Fluid was controlled with extracellular fluid, colloid solutions, and blood transfusions based on SVV monitoring. During surgery and for up to two hours after ICU admission, the following fluids were administered intravenously. The extracellular fluids were acetated Ringer’s solution containing 1% glucose (Physio®140) or bicarbonate Ringer’s solution (Bicanate®) (both Otsuka Pharmaceutical Factory, Inc., Tokushima, Japan). The colloid solutions were 6% HES 130/0.4/9 (Voluven®, Fresenius Kabi, Tokyo, Japan) and 5% albumin (donated albumin 5% IV injection 12.5 g/250 mL, JB Ltd., Japan Blood Products Organization, Tokyo, Japan). No glycine-containing fluids were administered. Transfusion exposure was recorded per routine, but the glycine content of blood components was not assayed.

Serum samples were frozen at -80℃ until required for assays. Glycine concentrations were measured using an enzyme-linked immunosorbent assay (Glycine ELISA Kit, K7013; Immundiagnostik AG, Bensheim, Germany) according to the manufacturer’s instructions.

Primary outcomes

Plasma glycine was measured in blood samples drawn at six time points: T0, before anesthesia induction; T1, 120 min after induction; T2, at completion of remifentanil administration; and T3, T4 and T5, at one, two and three hours, respectively, after completion of remifentanil administration. There are no prior perioperative data on glycine kinetics after remifentanil discontinuation. PAS typically emerges immediately in the post-anesthesia care unit and is short-lived, often resolving within the first 20-30 minutes; therefore, we obtained a sample at the end of remifentanil infusion (T2) to capture any peri-extubation change and then sampled hourly thereafter (T3-T5) to follow early postoperative dynamics [22]. For OIH, experimental human and randomized data indicate that hyperalgesia is maximal within the first postoperative hour and generally wanes by ~90-110 minutes after abrupt cessation of remifentanil, although persistence for several hours has been described; our 0-3-hour window was chosen to encompass this early peak and decay [23,24]. In addition, an intraoperative sample at 120 minutes after induction (T1) was included to represent a steady intraoperative exposure under remifentanil, providing a baseline for subsequent postinfusion changes.

Secondary outcomes

Perioperative shivering, Faces Pain Scale-revised (FPS-R, ©2001 International Association for the Study of Pain) [25], Confusion Assessment Method in the Intensive Care Unit (CAM-ICU, Copyright © 2002, E. Wesley Ely, MD, MPH, and Vanderbilt University, all rights reserved; formal written permission for text-only use was confirmed by the Vanderbilt CIBS Center; CAM-ICU materials were accessed from the official website, www.icudelirium.org) [26], and ionized magnesium concentration immediately after ICU admission were recorded. We selected the FPS-R (a six-face self-report pain scale scored on a 0-10 scale) for immediate post-anesthesia assessments to enable rapid self-report when early recovery factors (residual sedation, airway devices, delirium risk) can make the 0-10 numeric rating scale impractical. The FPS-R has been used in adult postoperative cohorts with acceptable convergent validity and sensitivity to change [27]. Delirium at ICU admission was assessed with the CAM-ICU using the standard four-feature algorithm (positive if Features 1 and 2 plus either 3 or 4). Ionized magnesium was measured by blood gas analyzer (Stat Profile pHOx® Ultra; Nova Biomedical, Waltham, MA) (reference range: 0.45-0.67 mmol/L).

Sample size

During planning of the study, we found few previous studies on plasma glycine during remifentanil infusion [20,21]. The number of cases was set at 12, based on the following two points and accounting for exclusions. 1) This is a preliminary study of plasma glycine levels and is subject to funding constraints. 2) A previous study of plasma glycine during remifentanil administration in the ICU included nine cases [20].

Statistical analysis

Nominal/ordinal variables were summarized as counts and percentages; continuous variables as mean ± SD. The primary analysis tested the within-subject time effect (T0-T5) on plasma glycine using a one-way repeated-measures ANOVA, followed by pre-specified Dunnett multiple comparisons versus T0 with family-wise error control. Normality of within-subject residuals was assessed using the Shapiro-Wilk test (with inspection of normal Q-Q plots). Sphericity was evaluated with Mauchly’s test; when violated, degrees of freedom and p-values were adjusted using the Greenhouse-Geisser epsilon (ε_GG), and GG-adjusted results are reported. Effect size for the time factor is presented as partial η². No additional covariates were included in the repeated-measures model. Analyses used complete-case data (subjects with all six time points), two-sided α = 0.05, and were conducted using JMP® 17 (JMP Statistical Discovery LLC, Cary, NC, USA).

## Results

Of the 12 patients enrolled, 10 completed the study. Two patients were excluded due to a change to revascularization surgery based on intraoperative findings. The patient characteristics are shown in Table 1.

**Table 1:** Patient characteristics PT-INR, prothrombin time-international normalized ratio; eGFR, estimated glomerular filtration rate Continuous data are shown as mean ± standard deviation, and categorical data as number (%). *eGFR was calculated using the equation devised by the Japanese Society of Nephrology [28].

Item	Value	Reference ranges
Age (years)	68 ± 8.7	Not applicable
Gender (female, %)	5 (50%)	Not applicable
Height (cm)	159 ± 6.2	Not applicable
Weight (kg)	52.8 ± 7.0	Not applicable
Body mass index (kg/m^2^)	20.4 ± 2.4	Not applicable
Aspartate transaminase (U/l)	36 ± 27	13-30
Alanine transaminase (U/l)	31 ± 22	10-42
Total bilirubin (mg/dL)	0.8 ± 0.4	0.4-1.5
Albumin (g/dL)	3.8 ± 0.7	4.1-5.1
PT-INR	1.0 ± 0.2	0.8-1.2
Creatinine (mg/dL)	0.7 ± 0.2	0.65-1.07
eGFR* (ml/min/1.73 m^2^)	74 ± 14	>60
Urea nitrogen (mmol/L)	16 ± 5.3	8-20

**Figure 1:**
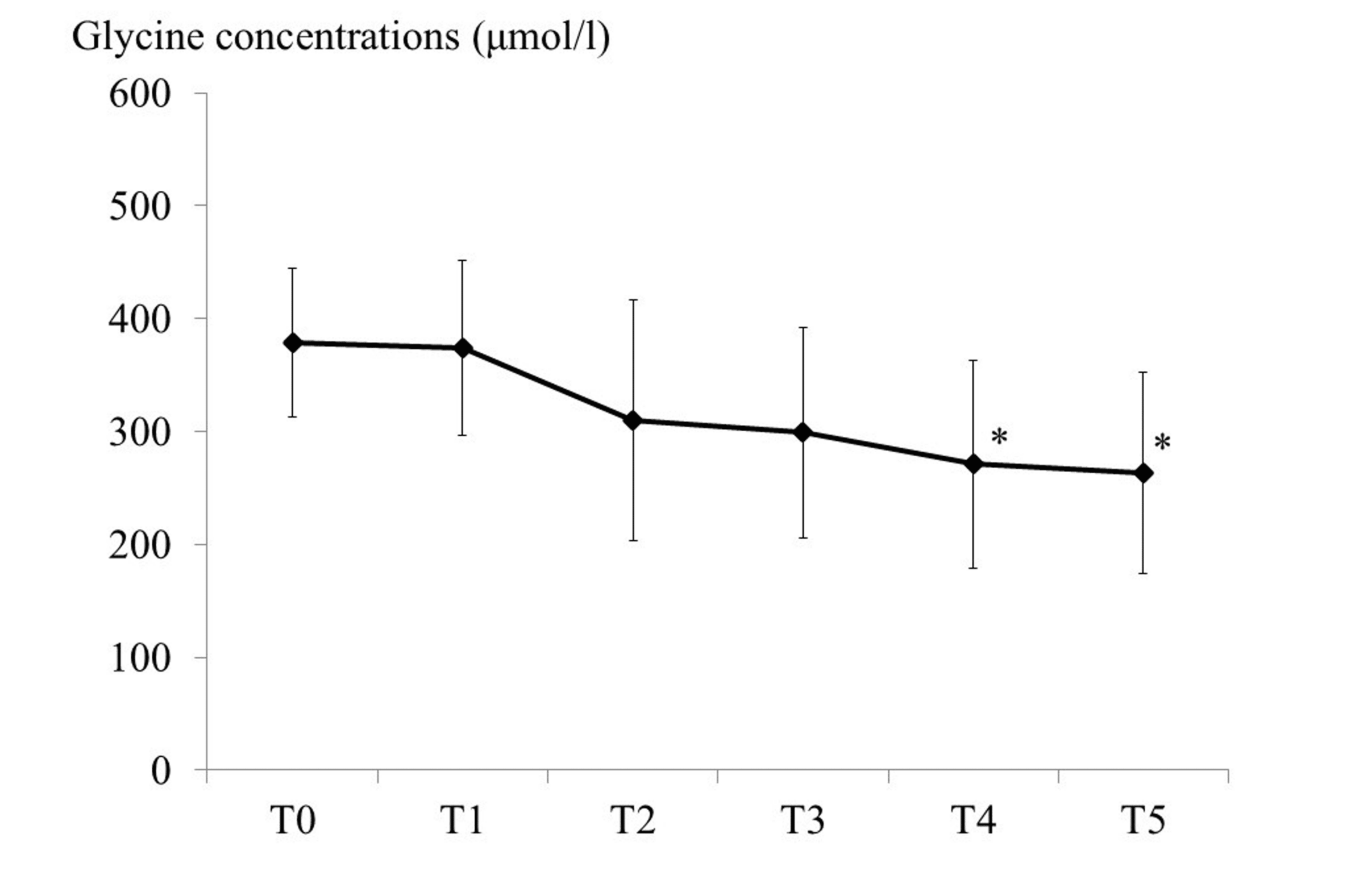
Time course of the plasma glycine concentration Time points: T0, before induction; T1, 120 min after induction; T2, completion of remifentanil administration; T3, T4, and T5, one, two, and three hours after completion of remifentanil administration, respectively. The reference range for plasma glycine is 148-438 μmol/L. Changes in plasma glycine were assessed by one-way repeated measures analysis of variance and a Dunnett post hoc test. P<0.05 was considered statistically significant. *Plasma glycine significantly decreased at T4 and T5 compared to T0.

As shown in Figure 1, plasma glycine tended to decrease over time despite remifentanil administration. In complete-case analyses (n = 10), a one-way repeated-measures ANOVA demonstrated a significant time effect after Greenhouse-Geisser (GG) correction (F_{1.53, 13.80} = 14.86, p_{GG} = 6.858×10⁻⁴). Model diagnostics indicated no material deviation from normality (Shapiro-Wilk W = 0.980, p = 0.235) and a violation of sphericity (Mauchly W = 0.157, χ²(df = 9) = 21.86, p = 0.009), for which GG correction (ε_GG = 0.383) was applied. Dunnett-adjusted pairwise comparisons versus T0 showed lower levels at T4 and T5 (adjusted p < 0.05), with no significant differences at T1-T3. The effect size for the time factor was large (partial η² ≈ 0.62).

**Figure 2:**
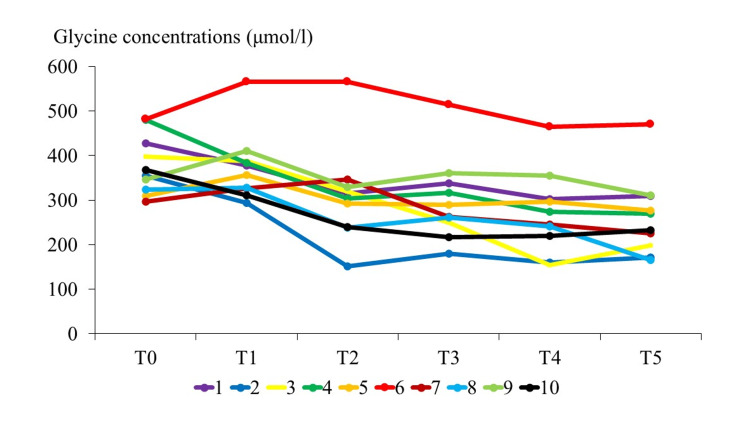
Time course of the plasma glycine concentration in each of the 10 patients Time points: T0, before induction; T1, 120 min after induction; T2, completion of remifentanil administration; T3, T4, and T5, one, two, and three hours after the completion of remifentanil administration, respectively. The reference range for plasma glycine is 148-438 μmol/L.

Changes in plasma glycine levels in the 10 patients are shown in Figure 2.

Only patient #6 had levels that exceeded the reference range (148-438 μmol/l). Intraoperative data are shown in Table 2 and secondary outcomes in the 10 patients are given in Table 3.

**Table 2:** Intraoperative data Data are shown as mean ± standard deviation.

Item	Value
Anesthesia time (minutes)	443 ± 92
Operation time (minutes)	370 ± 85
Blood loss (g)	765 ± 494
Urine output (ml)	635 ± 275
Fluid volume (ml)	3962 ± 934
Blood transfusion (ml)	180 ± 385
Body temperature (℃)	37 ± 0.6

**Table 3:** Secondary outcomes FPS-R, Faces Pain Scale-Revised (©2001 International Association for the Study of Pain) [25]. CAM-ICU, Confusion Assessment Method for the Intensive Care Unit (Copyright © 2002, E. Wesley Ely, MD, MPH, and Vanderbilt University, all rights reserved; formal written permission for text-only use was confirmed by the Vanderbilt CIBS Center; CAM-ICU materials were accessed from the official website, www.icudelirium.org) [26]. RBC, red blood cell; FFP, fresh frozen plasma * When epidural anesthesia was administered, it was performed between the eighth and ninth thoracic vertebrae. **Ionized magnesium was measured by Stat Profile pHOx® Ultra (Nova Biomedical, Waltham, MA) (reference range: 0.45-0.67 mmol/L)

Patient number	Remifentanil infusion rate (µg/kg/min)	Total dose of remifentanil (mg)	Glycine infusion rate (µg/kg/min)	Total dose of glycine (mg)	Total dose of fentanyl (μg)	Epidural anesthesia*	Shivering	FPS-R	CAM-ICU	Ionized magnesium** (mmol/L)	Blood transfusion
												1	0	0	0	0	350	Administered	None	0	Negative	0.56	None
2	0.05	1.3	0.375	9.75	400	Administered	Occurred	6	Negative	0.64	RBC 2 unit, FFP 4 unit
3	0.05	1.5	0.375	11.25	200	Administered	None	8	Negative	0.68	None
4	0.1	2	0.75	15	200	Administered	None	0	Negative	0.58	None
5	0.1	3.1	0.75	23.25	400	Administered	Occurred	6	Negative	0.51	None
6	0.1	1.1	0.75	8.25	0	Administered	None	2	Negative	0.56	None
7	0.15	2.2	1.125	16.5	150	Administered	None	2	Negative	0.59	None
8	0.15	4.5	1.125	33.75	800	None	None	6	Positive	0.61	RBC 4 unit, FFP 4 unit
9	0.2	5	1.5	37.5	700	None	None	0	Negative	0.56	None
10	0.2	3.6	1.5	27	800	Administered	None	6	Positive	0.55	None

The remifentanil infusion rates were not administered (n=1), 0.05 μg/kg/min (n=2), 0.1 μg/kg/min (n=2), 0.15 μg/kg/min (n=3), and 0.2 μg/kg/min (n=2). Epidural anesthesia was given at Th 8/9 but was not used in two patients. Shivering occurred in two patients and CAM-ICU was positive in two patients.

## Discussion

Measurement of plasma glycine levels during and after remifentanil administration showed no significant increase at any infusion rate. Compared with the level immediately after induction (T0), plasma glycine was significantly lower at two hours (T4) and three hours (T5) after discontinuation of remifentanil. Overall, plasma glycine ranged from 200 to 500 μmol/L. In one case, the concentration exceeded the reference range (148-438 μmol/L).

In ICU patients, Bonnet et al. reported rising plasma glycine levels during infusion when using a remifentanil formulation containing 3 mg of glycine per 1 mg of remifentanil [20]. In contrast, despite our use of a formulation with a higher glycine content (7.5 mg per 1 mg remifentanil), we did not observe an increase. A survey of perioperative amino acid levels in patients undergoing pancreatic cancer surgery likewise found no significant change in glycine levels [29]. Differences in patient selection likely contributed: our cohort excluded overt hepatic and renal dysfunction, whereas the ICU cohort included substantial organ impairment.

Inter-individual differences in perioperative glycine may reflect factors beyond remifentanil exposure, including baseline nutritional status and the catabolic/inflammatory response to major surgery, which can lower circulating glycine postoperatively [30], as well as latent variability in hepatic glycine metabolism (glycine-cleavage system activity) [11], hepatic conjugation flux [12], and renal clearance [20,21]. These mechanisms may also help explain the divergent behavior across studies and within our cohort. In patient #6, the plasma glycine level exceeded the reference range, but there was no evidence of hepatic or renal dysfunction and the remifentanil infusion rate was only 0.1 μg/kg/min (total dose: 1.1 mg). Thus, there was no obvious cause for the elevation. Also, no apparent signs of OIH or PAS were observed. However, in such patients, administration of higher concentrations of remifentanil or concomitant use of glycine-containing products (e.g., rocuronium preparations containing glycine as a preservative) may cause plasma glycine to significantly exceed normal levels, potentially further elevating the CSF glycine level.

This study has several limitations. First, it was a single-center investigation restricted to one surgical procedure (pancreaticoduodenectomy) and a limited, standardized anesthetic regimen, which may restrict generalizability. Second, patients with hepatic or renal dysfunction were excluded, limiting external validity in populations with altered glycine metabolism or clearance. Third, this was a preliminary study with a small sample size (n=10 completers), which reduced precision. Moreover, the failure of glycine concentrations to increase precluded a robust assessment of the concentration-infusion-rate relationship as described by Bonnet et al. [20], and direct evaluation of the NMDA-mediated mechanism linking remifentanil-derived glycine to PAS/OIH was not possible. Fourth, the mechanistic hypothesis presupposes a rise in circulating glycine sufficient to engage NMDA-receptor plausibly mediated effects; however, plasma glycine did not increase in 9 of 10 participants, effectively preventing a direct test in this dataset. Further research involving larger cohorts, particularly those exposed to higher cumulative glycine levels (e.g., higher remifentanil doses or co-administration of glycine-containing formulations), is required to determine whether plasma glycine concentration rises during the perioperative period and to enable formal modeling. Fifth, potential glycine exposure from transfused blood components was not quantified. While standard red cell additive solutions are glycine-free, residual donor plasma and the supernatant of packed red blood cells may contain amino acids, including glycine. Thus, transfusion could have contributed a small, unmeasured glycine load in some cases. In this preliminary study, we did not perform transfusion-stratified analyses.

## Conclusions

Plasma glycine measured during remifentanil administration did not increase significantly at any infusion rate in 9 of 10 cases, precluding a meaningful test of our NMDA-mediated mechanistic hypothesis. Larger cohorts and settings involving higher cumulative glycine exposure are required to establish whether plasma glycine concentration rises during surgery and to evaluate any relationship between concentration and infusion rate.
